# Quantitative CT analysis to predict esophageal fistula in patients with advanced esophageal cancer treated by chemotherapy or chemoradiotherapy

**DOI:** 10.1186/s40644-022-00490-2

**Published:** 2022-11-04

**Authors:** Yan-Jie Shi, Chang Liu, Yi-Yuan Wei, Xiao-Ting Li, Lin Shen, Zhi-Hao Lu, Ying-Shi Sun

**Affiliations:** 1 Key Laboratory of Carcinogenesis and Translational Research (Ministry of Education), Department of Radiology, Peking University Cancer Hospital & Institute, No.52 Fu Cheng Road, Hai Dian District, Beijing, 100142 China; 2 Key Laboratory of Carcinogenesis and Translational Research (Ministry of Education), Department of Gastrointestinal Oncology, Peking University Cancer Hospital & Institute, No.52 Fu Cheng Road, Hai Dian District, Beijing, 100142 China

**Keywords:** Esophageal squamous cell cancer, X-ray computed tomography, Esophageal fistula, Risk factor, Chemoradiotherapy

## Abstract

**Background:**

Esophageal fistula is one of the most serious complications of chemotherapy or chemoradiotherapy (CRT) for advanced esophageal cancer. This study aimed to evaluate the performance of quantitative computed tomography (CT) analysis and to establish a practical imaging model for predicting esophageal fistula in esophageal cancer patients treated with chemotherapy or chemoradiotherapy.

**Methods:**

This study retrospectively enrolled 204 esophageal cancer patients (54 patients with fistula, 150 patients without fistula) and all patients were allocated to the primary and validation cohorts according to the time of inclusion in a 1:1 ratio. Ulcer depth, tumor thickness and length, and minimum and maximum enhanced CT values of esophageal cancer were measured in pretreatment CT imaging. Logistic regression analysis was used to evaluate the associations of CT quantitative measurements with esophageal fistula. Receiver operating characteristic curve (ROC) analysis was also used.

**Results:**

Logistic regression analysis showed that independent predictors of esophageal fistula included tumor thickness [odds ratio (OR) = 1.167; *p* = 0.037], the ratio of ulcer depth to adjacent tumor thickness (OR = 164.947; *p* < 0.001), and the ratio of minimum to maximum enhanced CT value (OR = 0.006; *p* = 0.039) in the primary cohort at baseline CT imaging. These predictors were used to establish a predictive model for predicting esophageal fistula, with areas under the receiver operating characteristic curves (AUCs) of 0.946 and 0.841 in the primary and validation cohorts, respectively. The quantitative analysis combined with T stage for predicting esophageal fistula had AUCs of 0.953 and 0.917 in primary and validation cohorts, respectively.

**Conclusion:**

Quantitative pretreatment CT analysis has excellent performance for predicting fistula formation in esophageal cancer patients who treated by chemotherapy or chemoradiotherapy.

**Supplementary Information:**

The online version contains supplementary material available at 10.1186/s40644-022-00490-2.

## Introduction

Latest cancer statistics revealed that esophageal cancer was an extremely devastating disease among the 10 most common malignancies causing death [1]. According to the Comprehensive Registry of Esophageal Cancer in Japan, the incidence of T4b disease represents approximately 9% of all thoracic esophageal cancer [2]. The incidence of distant metastases is approximately 40% among all esophageal cancer patients [1]. Curative resection is not feasible in esophageal cancer patients with T4b or distant metastasis, which means that those patients have to face unfavorable prognosis [2]. Chemotherapy or chemoradiotherapy (CRT) without planned esophagectomy are the most attractive treatment options available for unresectable esophageal cancer [3]. Previous reports indicated that chemotherapy or CRT could allow 32–60% of patients to undergo curative resection for cT4b esophageal cancer, suggesting that curative resection could provide a good prognosis [4–6].

However, esophageal fistula is one of the known serious complications of esophageal cancer, especially in cases treated by chemotherapy or CRT. Chemotherapy or CRT could induce esophageal fistula by damaging the walls of the esophagus and adjacent organs. The imbalance between the treatment response of the tumor and normal tissue repair may lead to esophageal fistula [7–9]. Although the incidence of esophageal fistula is low with a range of 10.4–13.9%, it is potentially life-threatening with high mortality rates of 10–25% [10, 11]. It is generally admitted that mortality could at least double when the diagnostic and therapeutic delay exceeds 24 hours [12]. Therefore, esophageal fistula could not only result in a poor quality of life, but also changes the therapeutic effect and clinical management.

Thus, predicting esophageal fistula occurrence and identifying the associated high-risk factors are major clinical problems before chemotherapy or CRT for esophageal carcinoma. Various risk factors have been considered key factors associated with esophageal fistula formation in patients with esophageal cancer after treatment, e.g., esophageal stenosis, nutritional status, body mass index (BMI), tumor length, presence of ulcer in the tumor and T stage [13–15].

Currently, chest computed tomography (CT), endoscopic ultrasound (EUS), endoscopy and esophagography are the main imaging tools for evaluating esophageal cancer. EUS are limited in evaluating advanced esophageal tumors whose outer borders might be outside the field of view, especially stenotic tumors [16]. Endoscopy and EUS are direct and effective methods, but both highly operator-dependent, could damage the esophagus, which may lead to esophageal fistula in high risk patients with suspicious esophageal fistula. Meanwhile, chest CT is inexpensive, easy to perform and reproducible, and could provide morphological and quantitative information for the lesions and surrounding conditions [17]. Therefore, CT may provide useful information for predicting esophageal fistula before treatment.

To our knowledge, the role of CT features in predicting esophageal fistula had been rarely reported. Therefore, this retrospective study aimed to identify risk factors for esophageal fistula, to investigate the performance of quantitative CT analysis and to develop a practical imaging model in predicting the risk of esophageal fistula before treatment in patients with esophageal cancer.

## Methods

### Patients

Inclusion criteria were: a) gastroscopic biopsy-proven esophageal squamous cell carcinoma (SCC) before treatment; b) chest enhanced CT examination at baseline; c) patients treated with chemotherapy or CRT; d) assessing whether esophageal fistula was presented after treatment by CT, endoscopy, barium esophagography or operation at follow-up of 6 months; e) availability of quality diagnostic images for measuring lesions. Patients who were not in accord with inclusion criteria were excluded. Patients with esophageal cancer accompanied by esophageal fistula after chemotherapy or CRT were identified from January 2011 to December 2019. In addition, esophageal SCC patients with no esophageal fistula after chemotherapy or CRT were included in this study from January 2016 to December 2017 in a 1:3 ratio of non-fistula and fistula groups. A total of 204 patients were enrolled in this study. Totally 54 patients with esophageal fistula were identified. In parallel, 150 esophageal SCC patients with non-esophageal fistula after treatment were included in this study. The patients were allocated to the primary and validation cohorts in a 1:1 ratio according to the time of inclusion. The first 102 patients (27 patients with fistula and 75 patients without fistula) were allocated to the primary cohort, and the subsequent 102 patients (27 patients with fistula and 75 patients without fistula) were allocated to the validation cohort. The complete patient enrollment process is shown in Supplementary Fig. 1.

### Chemotherapy or CRT

Patients treated with chemotherapy received a platinum-based regimen, mainly including paclitaxel (175 mg/m^2^ i.v. on day 1 of every 3-week cycle) and cisplatin (75 mg/m^2^ i.v. on day 1 of every 3-week cycle). Other patients were treated with concurrent chemoradiotherapy, with a radiotherapy dose of 50-60Gy and platinum-based chemotherapy.

### Computed tomography

All patients received enhanced multi-slice CT (MDCT) scanning of the chest before and during chemotherapy or CRT. Scans were performed using a 64-row helical CT scanner (Lightspeed VCT; General Electrical Medical Systems, Milwaukee, WI, USA). All patients were in the supine position. Generally, the scan began at 2.0 cm above the lung apices and extended through the adrenal glands. The following imaging parameters were used: 120–140 kVp tube voltage; 300 mA tube current; 64 × 0.625 mm detector collimation; 0.6 s/gantry rotation speed, and 1.5 helical pitch. Axial, coronal and sagittal images were reconstructed using a section width of 5.0 mm. One hundred milliliters of the non-ionic contrast medium iohexol (Omnipaque 300; GE Healthcare) were injected at a rate of 3.0 mL/s through the median cubital vein. The enhanced CT scan was performed 55 s after the start of the contrast medium injection.

### Image interpretation

MDCT imaging data was transferred to a picture archiving and communication system (PACS). Two radiologists with 8 years (Dr. Wei) and 12 years (Dr. Shi) of experience in thoracic CT independently reviewed the axial and reconstructed CT images obtained at baseline. Both reviewers were blinded to final results about esophageal fistula. All qualitative and quantitative parameters were assessed on enhanced images before treatment. Final quantitative measurements were determined by averaging the values obtained by the two radiologists. For qualitative analysis, the diagnosis was confirmed by a third experienced radiologist in case of disagreement.

### Qualitative analysis

**Fig. 1:**
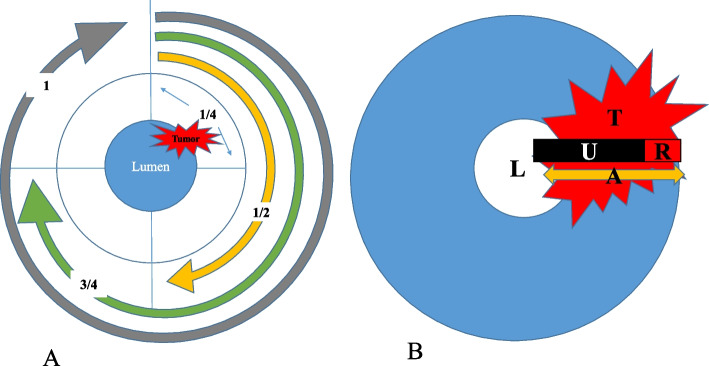
Schematic diagram of esophageal carcinoma range and measurement of a deep ulcer. **A** Schematic diagram demonstrated that the esophageal lumen was evenly divided into four parts. The tumor (red) range was diagnosed as 0–1/4 (two blue arrow), 1/4–1/2 (yellow arc), 1/2–3/4 (green arc) and 3/4–1 (grey arc). **B** Schematic diagram demonstrated how ulcer depth (U, black), residual esophageal wall in the ulcer layer (R, red) and the the thickness of the lesion adjacent to ulcer (A, yellow) were measured

Tumor locations were classified as cervical, upper thoracic, middle thoracic, or lower thoracic esophagus. An important factor in assessing cancer location was to determine the center of the tumor in the esophagus. The tumor was staged by MDCT before therapy according to AJCC/TNM classification, 8th edition. The MDCT status was defined as follows [18]: CT T0, wall thickness < 5.0 mm and no signs of adventitial penetration; CT T1–2, a wall thickness of at least 5–10 mm without evidence of adventitial penetration; CT T3, tumor exhibiting a wall thickness of > 10 mm, possibly appearing as ill-defined, abnormal soft tissue around the tumor but no invasion of adjacent structures; CT T4a, invasion of the pleura, pericardium and diaphragm; T4b, invasion of the aorta, vertebral body and trachea. In the last two stages, the tumor had a wall thickness of > 10 mm and invaded adjacent structures. Intrathoracic and abdominal lymph nodes > 10 mm and supraclavicular lymph nodes > 5.0 mm in short-axis diameter were considered metastatic lymph nodes [18]. N staging was classified as negative (N-) or positive (N+) metastatic lymph nodes. CT imaging findings related to tracheal or bronchial invasion of the tumor were analyzed. The tumor range was classified as four types, including 0–1/4, 1/4–1/2, 1/2–3/4 and 3/4–1 (Fig. 1). The range of esophageal cancer was evenly classified as four types. If the esophageal tumor occupied up to a quarter of the esophageal wall, the range of tumor would be defined as type of 0–1/4. If the tumor occupied a quarter to a half of the esophageal wall, the tumor range would be defined as type of 1/4–1/2. Similarly, if the tumor occupied a half to three quarters of the esophageal wall, the tumor range would be defined as type of 1/2–3/4. Then, if the tumor occupied three quarters to whole of the esophageal wall, the tumor range would be defined as type of 3/4–1. Esophageal stenosis and deep ulcer were also evaluated. The morphological patterns of the tumor were graded as focal or diffuse type.

### Quantitative analysis

**Fig. 2:**
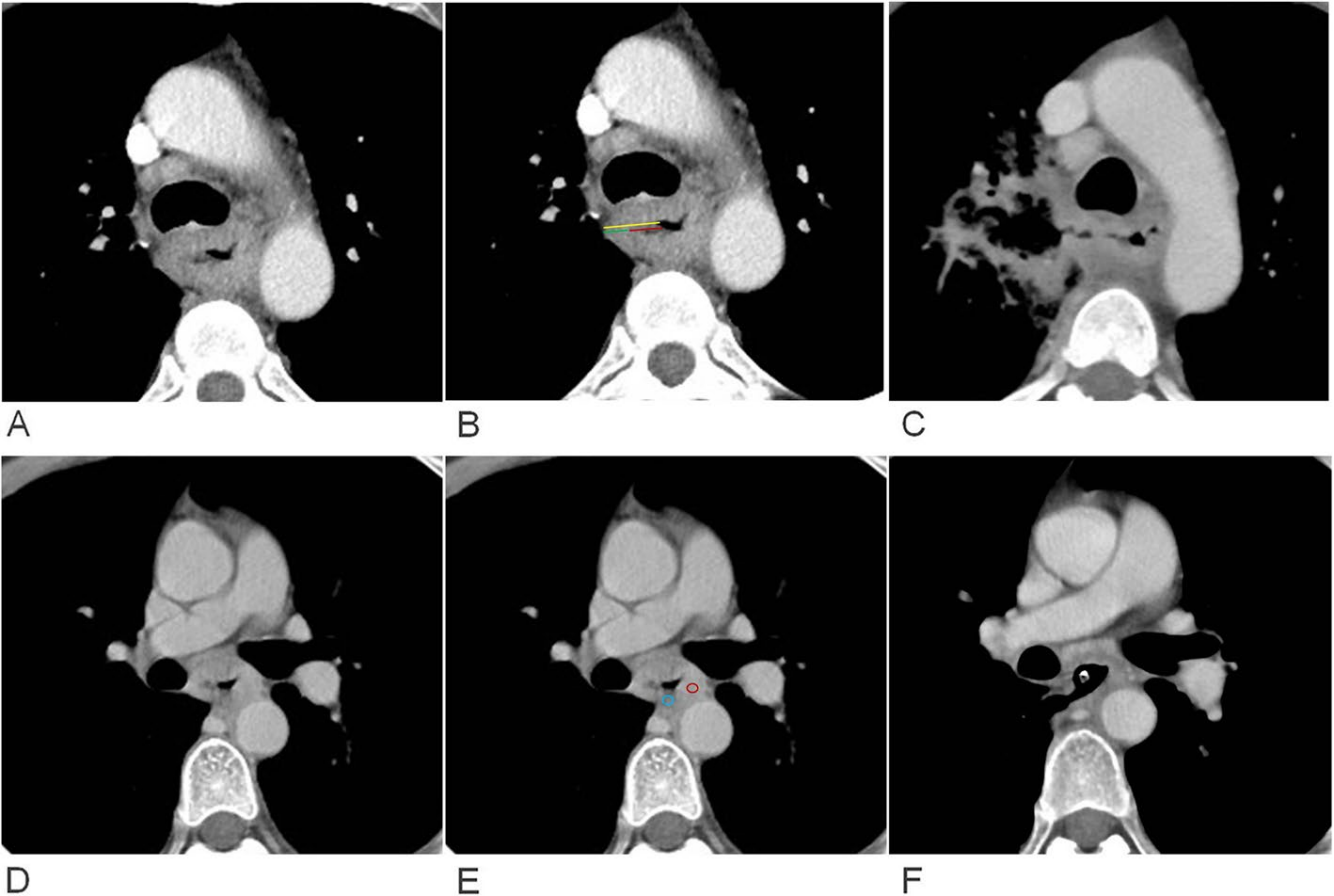
Axial enhanced CT at baseline examination showing ROI delineation and deep ulcer measurement. (A-C) CT images in a 42-year-old woman with esophageal cancer. **A** CT image showed an esophageal mass with a deep ulcer in the right wall. **B** The same CT image showed the measurement of the depth of deep ulcer (red line) at 10 mm, residual esophageal wall (green line) at 7 mm and the thickness of adjacent wall to ulcer (yellow line) at 17 mm in the ulcer layer. The ratio of ulcer depth to adjacent tumor thickness of 58.8% and tumor thickness of 17 mm could predict fistula formation after treatment. **C** The esophageal fistula occurred in the region of the deep ulcer after three chemotherapy cycles and radiotherapy. **D**-**F** CT images in a 50-year-old man with esophageal cancer. **D** CT image showed an esophageal mass with a thickness of 15 mm. **E** The highest (red circle; 119 HU) and lowest enhancement (blue circle; 20 HU) zones, the ratio of minimum to maximum enhanced CT value of 16.8% and a tumor thickness of 15 mm could predict fistula formation after treatment. **F** Esophageal fistula occurred in the region of the lowest zone after three chemotherapy cycles and radiotherapy

Tumor wall thickness (THK-tumor) of esophageal SCC was measured perpendicularly to the lumen on axial images using the workstation’s electronic caliper. If the lumen was not visible in esophageal cancer with diffuse type, thickness of tumor was obtained through the following method. The entire diameter of the esophagus including the invisible lumen and the tumor was measured, and then multiplied by a factor of 0.5. The above method had been used in some researches [18, 19]. When the lumen was not visible in esophageal cancer with focal or eccentric type, the boundary of tumor in the lumen side was defined through observing sagittal and coronal images by radiologists and then maximal thickness was measured [18, 19]. Tumor length (the longest diameter of tumor, L-tumor) was measured on sagittal CT images. The region of interest (ROI) of the maximum CT value (HU-max) of tumor was placed on the highest enhanced area and that of the minimum CT value (HU-min) of tumor on the lowest enhanced area (Fig. 2). The area of each ROI was 3–5 mm^2^, averaging three measurements. The depths of low and high intensity enhancement areas were also measured (Fig. 2). Tumor ulceration was quantitatively assessed by measuring ulcer depth (DEP-ulcer), the thickness of the residual wall in the ulcer layer (THK-residue), and the thickness of the lesion adjacent to the ulcer (THK-adjacency) on cross-sectional CT images (Figs. 1 and 2). In case of no ulcer in the tumor, DEP-ulcer was recorded as 0, and THK-residue and THK-adjacency were the same as the wall thickness of the tumor. The ulcer-to-tumor ratio (R-ulcer) was calculated by the following equation: DEP-ulcer/THK-adjacency. The THK-residue-to-tumor ratio (R-residue) was derived as THK-residue/THK-adjacency. The HU-min to HU-max (R-HU) ratio was obtained as HU-min/HU-max. The joint predictive efficiency of continuous variables was defined as Y1. Y1 combining qualitative signs were defined as Y2.

### Definition of esophageal fistula

Esophageal fistula was defined as a connection between the esophagus and adjacent organs or tissues [20] detected by CT, endoscopy, esophagography or operation. On CT images, esophageal fistula was diagnosed by discontinuous or defective esophageal wall, gas and fluid accumulated around the esophagus, or pneumonia associated with esophageal fistula. By esophagography, esophageal fistula was identified as contrast medium leakage into the mediastinum or bronchus.

### Statistical analysis

Differences in qualitative parameters in patients with esophageal SCC between the primary and validation cohorts were assessed by the Mann-Whitney test or the Chi-square test/Fisher’s exact test. Differences in quantitative factors were examined by independent-samples t test or the Mann-Whitney test. The associations of quantitative measurements were evaluated by Pearson correlation coefficient; a coefficient > 0.6 suggested a moderate or strong correlation. Only parameters with weak correlation were substituted into the multivariate equation. Receiver operating characteristic (ROC) curve analysis was applied to evaluate the predictive capability of the quantitative analysis for predicting esophageal fistula, with the area under the ROC curve (AUC). Intraclass correlation coefficients (ICCs) were determined to evaluate inter-observer agreement in terms of CT quantitative measurements. Data analysis was conducted with SPSS 22.0 (IBM Corporation, Armonk, NY, USA) and STATA 12.0 (Stata Corporation, College Station, TX, USA).

## Results

### Clinical characteristics of the patients

The clinical characteristics of the patients are summarized in Table 1. There were no significant differences in clinical characteristics between the primary and validation cohorts (See Supplementary Table 1). Two radiologists independently assessed esophageal SCC, achieving satisfactory agreement. Qualitative and quantitative analyses between the two radiologists showed perfect or substantial agreement with ICCs of 0.79–0.96 (See Supplementary Table 2). The ICC values of the detection of ulcer (qualitative) and the measurement of ulcer depth (quantitative) between two readers were 0.85 and 0.89, indicating perfect inter-observer agreement.

**Table 1:** Characteristics of patients in the primary and validation cohorts

Characteristic	Primary cohort		***P***	Validation cohort		***P***
	non-fistula	fistula		non-fistula	fistula	
Gender (%)			0.185			0.307
Male	68 (90.67)	27 (100)		69 (92)	23 (85.19)	
Female	7 (9.33)	0 (0)		6 (8.0)	4 (14.81)	
Age, mean ± SD, years	60.62 ± 8.05	59.07 ± 7.26	0.38	60.54 ± 8.04	58.78 ± 7.82	0.353
Grade (%)			0.125			0.881
Mid-high	43 (57.33)	20 (74.07)		46 (61.33)	17 (62.96)	
Low	32 (42.67)	7 (25.93)		29 (38.67)	10 (37.04)	
Location (%)			0.004			0.09
Neck	4 (5.33)	1 (3.70)		6 (8.0)	1 (3.70)	
Upper-thorax	14 (18.67)	2 (7.41)		14 (18.67)	6 (22.22)	
Mid-thorax	29 (38.67)	21 (77.78)		31 (41.33)	17 (62.96)	
Low-thorax	28 (37.33)	3 (11.11)		24 (32.0)	3 (11.11)	
Treatment (%)			0.822			0.610
Chemotherapy	37 (49.33)	14 (51.85)		32 (42.67)	10 (37.04)	
Chemoradiotherapy	38 (50.67)	13 (48.15)		43 (57.33)	17 (62.96)	
T stage (%)			< 0.001			0.059
T1	0 (0)	0 (0)		0 (0)	0 (0)	
T2	9 (12)	0 (0)		7 (9.33)	1 (3.70)	
T3	61 (81.33)	12 (44.45)		59 (78.67)	17 (62.97)	
T4a	0 (0)	1 (3.70)		1 (1.33)	3 (11.11)	
T4b	5 (6.67)	14 (51.85)		8 (10.67)	6 (22.22)	
Trachea invasion (%)			< 0.001			0.5
No	73 (97.33)	15 (55.56)		67 (89.33)	23 (85.19)	
Yes	2 (2.67)	12 (44.44)		8 (10.67)	4 (14.81)	
N stage (%)			0.321			0.021
No	5 (6.67)	0 (0)		8 (10.67)	0 (0)	
Yes	70 (93.33)	27 (100)		67 (89.33)	27 (100)	
Metastasis (%)			0.387			0.55
No	60 (80)	24 (88.89)		62 (82.67)	24 (88.89)	
Yes	15 (20)	3 (11.11)		13 (17.33)	3 (11.11)	
Tumor range(%)			< 0.001			0.025
0–1/4	1 (1.34)	0 (0)		0 (0)	0 (0)	
1/4–1/2	18 (24.0)	0 (0)		11 (14.67)	1 (3.70)	
1/2–3/4	25 (33.33)	2 (7.41)		30 (40.0)	6 (22.22)	
3/4–1	31 (41.33)	25 (92.59)		34 (45.33)	20 (74.07)	
Type (%)			0.195			0.952
Focal	17 (22.67)	3 (11.11)		19 (25.33)	7 (25.93)	
Diffuse	58 (77.33)	24 (88.89)		56 (74.67)	20 (74.07)	
Luminal obliteration (%)			0.303			0.634
No	25 (33.33)	12 (44.44)		26 (34.67)	8 (29.63)	
Yes	50 (66.67)	15 (55.56)		49 (65.33)	19 (70.37)	
Deep ulcer(%)			< 0.001			< 0.001
No	70 (93.33)	5 (18.52)		66 (88.0)	9 (33.33)	
Yes	5 (6.67)	22 (81.48)		9 (12.0)	18 (66.67)	
THK-tumor (mm)	14.73 ± 4.99	17.89 ± 5.07	0.006	15.29 ± 6.33	17.44 ± 5.90	0.118
L-tumor (mm)	54.20 ± 20.86	81.67 ± 26.19	< 0.001	54.73 ± 22.97	75.19 ± 28.15	< 0.001
DEP-ulcer (mm)	0.61 ± 2.40	10.46 ± 6.89	< 0.001	0.96 ± 2.73	6.59 ± 5.73	< 0.001
THK-residue (mm)	14.16 ± 5.20	9.54 ± 3.96	< 0.001	13.91 ± 6.76	9.70 ± 7.86	0.002
THK-adjacency (mm)	14.59 ± 4.83	16.54 ± 4.96	0.082	14.93 ± 6.20	15.63 ± 5.85	0.392
R-ulcer (%)	5.07 ± 19.65	68.98 ± 52.67	< 0.001	6.42 ± 18.21	46.12 ± 37.47	< 0.001
R-residue (%)	96.67 ± 13.07	43.75 ± 28.42	< 0.001	93.15 ± 19.08	59.01 ± 33.54	< 0.001
HU-min (HU)	43.86 ± 16.34	26.33 ± 13.55	< 0.001	45.17 ± 17.32	30.52 ± 13.15	< 0.001
THK-min (mm)	11.13 ± 4.55	13.93 ± 4.66	0.003	11.35 ± 5.29	12.41 ± 5.21	0.202
R-min	0.77 ± 0.19	0.79 ± 0.17	0.939	0.75 ± 0.17	0.74 ± 0.25	0.802
HU-max (HU)	93.75 ± 14.29	90.53 ± 12.75	0.286	94.28 ± 15.45	85.61 ± 12.89	0.006
THK-max (mm)	11.15 ± 3.81	13.89 ± 4.93	0.01	10.88 ± 3.78	12.00 ± 2.76	0.068
R-HU	0.47 ± 0.17	0.30 ± 0.14	< 0.001	0.48 ± 0.17	0.36 ± 0.15	0.002

*Abbreviations*: *N stage* Lymph node stage, *THK-tumor*, Tumor thickness, L*-tumor* Tumor length, *DEP-ulcer*, Depth of deep ulcer, *THK-residue* Thickness of residual esophageal wall in the ulcer layer, *THK-adjacency* Thickness of lesion adjacent to the ulcer, *R-ulcer* The ulcer-to-tumor ratio, *R-residue* THK-residue-to-tumor ratio, *HU-min* Tumor minimum CT value, *THK-min* Thickness of the tumor on minimum CT value layer, *R-min* THK-min-to-THK-tumor ratio, *HU-max* Tumor maximum CT value, *THK-max* Thickness of the tumor on maximum CT value layer, *R-HU* HU-min-to-HU-max ratio

### Univariable comparisons of CT’s quantitative parameters

Table 1 shows the univariable comparisons of qualitative and quantitative CT parameters for predicting fistula in the primary and validation cohorts. The qualitative analysis showed that T4b staging, trachea invasion, tumor range of 3/4–1, tumor location in the middle thoracic esophagus and ulcer presence were risk factors for esophageal fistula before treatment (*P* < 0.05). There were significant differences in tumor location, range, and presence of deep ulcers between the fistula and non-fistula groups in qualitative analysis (*P* < 0.001). In quantitative analysis, tumor thickness, tumor length, depth of the ulcer and ulcer ratio in the fistula group were larger than those of the non-fistula group (*P* < 0.001). HU-min, and ratio of HU-min to HU-max were smaller in the fistula group compared with the non-fistula group in the primary and validation cohorts (*P* < 0.05).

### Predictive performance of CT’s quantitative analysis

Correlation analyses of parameters obtained in CT analysis revealed that the correlation coefficients for HU-min and HU-min to HU-max ratio, THK-tumor and THK-adjacency, and presence of ulcer, DEP-ulcer and R-ulcer were > 0.6. HU-min to HU-max ratio, THK-tumor and R-ulcer were used in logistic regression model for predicting esophageal fistula due to high AUCs.

This logistic regression analysis revealed that esophageal cancer with high R-ulcer had elevated predicted rate of esophageal fistula [odds ratio (OR) = 164.947; 95% confidence interval (CI): 20.464–1329.511]. Esophageal cancer with lower R-HU value was associated with higher rate of esophageal fistula after treatment (OR = 0.006; 95% CI: 0.001–0.782). Esophageal cancer with higher THK-tumor was also correlated with higher rate of esophageal fistula (OR = 1.167; 95% CI: 1.009–1.351) (Table 2). R-ulcer had the highest performance with an AUC of 0.887 (95%CI 0.800–0.975) for predicting esophageal fistula, followed by R-HU with an AUC of 0.774 (95%CI 0.677–0.872) and THK-tumor with an AUC of 0.696 (95%CI 0.582–0.809) (Table 2).

**Table 2:** Logistic regression analysis of quantitative variables in the primary cohort

Variables	B	OR	95%CI	*P*	AUC	Cutoff
THK-tumor	0.155	1.167	1.009–1.351	0.037	0.696 (0.582–0.809)	15.5^a^
R-ulcer	5.106	164.947	20.464–1329.511	< 0.001	0.887 (0.800–0.975)	0.18^a^
R-HU	-5.105	0.006	0.001–0.782	0.039	0.774 (0.677–0.872)	0.37

*Abbreviations*: *THK-tumor* Tumor thickness, *R-ulcer*, The ulcer-to-tumor ratio, *R-HU* HU-min-to-HU-max ratio, *B* regression coefficient, *OR* Odds ratio, *95%CI* 95%confidence interval, *AUC* Area under the curve

^a^the value >cutoff value indicated oesophageal fistula

**Fig. 3:**
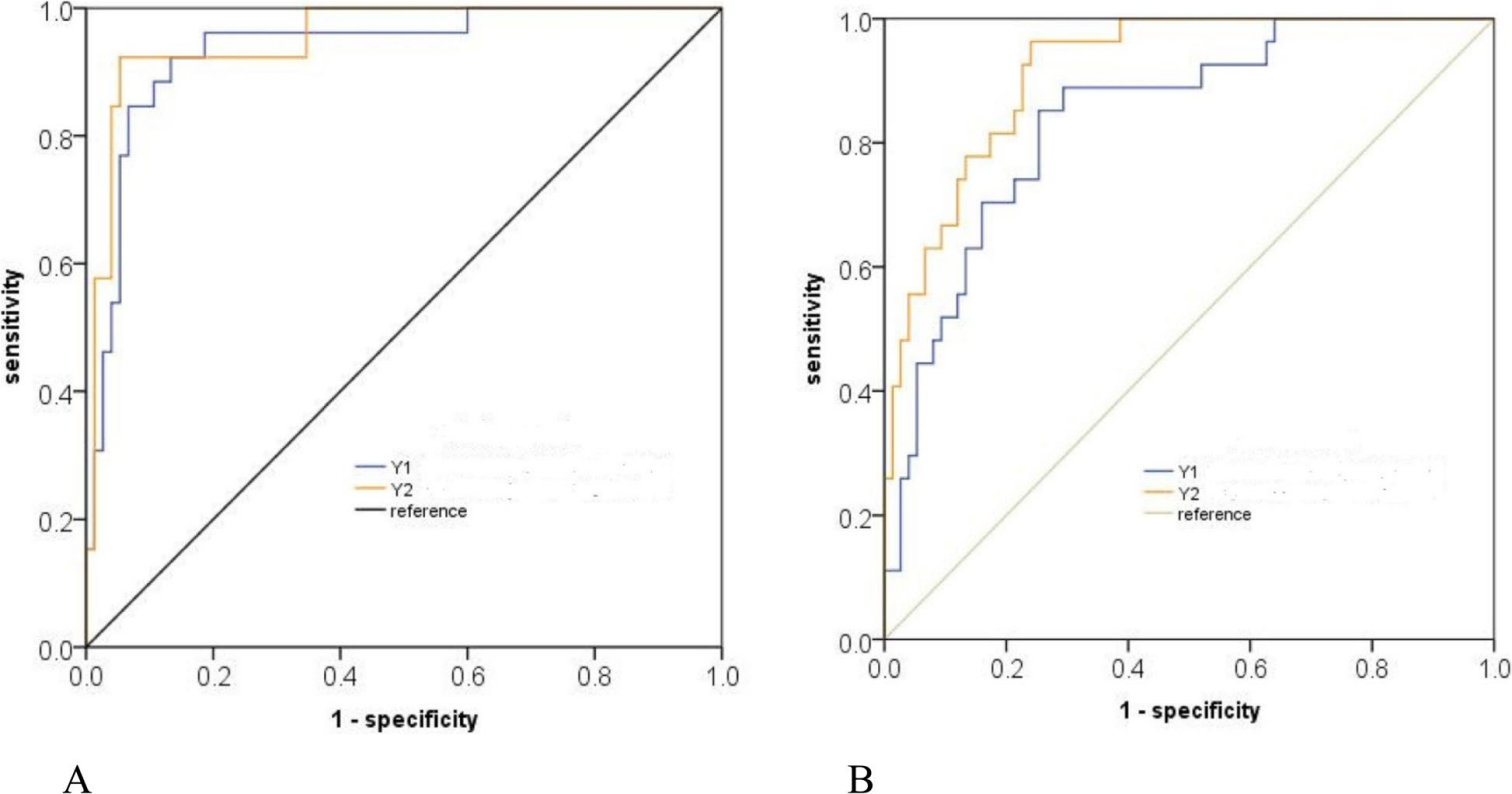
ROCs of baseline CT for predicting esophageal fistula in the primary and validation cohorts. The blue line (Y1) depicted the predictive performance of quantitative CT analysis. The yellow line (Y2) depicted the performance of quantitative analysis combined with T stage. The AUCs for Y1 and Y2 were 0.946 and 0.953 in the primary cohort (**A**) and 0.841 and 0.917 in the validation cohort (**B**), respectively (both *p* > 0.05)

So, R-ulcer, R-HU and THK-tumor were used for establishing logistic regression model due to high performance in predicting esophageal fistula at baseline CT. This quantitative CT model (using the formula Y1 = 0.155*THK-tumor+ 5.106*R-ulcer-5.105*R-HU) for predicting esophageal fistula had great performance with an AUC of 0.946 (95%CI 0.902–0.990) and an accuracy of 92.2% in the primary cohort. The same high performance was found in the validation cohort with an AUC of 0.841 (95%CI 0.758–0.924) and an accuracy of 78.4% in predicting esophageal fistula. Detailed information of the efficiency in predicting esophageal fistula in the primary and validation cohorts was shown in Table 3. The predictive capabilities of quantitative CT model for esophageal fistula in the primary and validation cohorts were determined by ROC curve analysis (Fig. 3).

**Table 3:** The performance of qualitative and quantitative variables for predicting esophageal fistula in the primary and validation cohorts

Cohorts	Variables	AUC	Cutoff	SEN	SPE	PPV	NPV	ACU
Primary cohort	Y1	0.946 (0.902–0.990)	2.1^a^	0.885	0.933	0.828	0.959	0.922
	Y2	0.953 (0.909–0.997)	2.58^a^	0.923	0.947	0.862	0.973	0.941
	Trachea invasion	0.709 (0.579–0.838)	Yes	0.444	0.973	0.857	0.839	0.833
	Tumor range	0.756 (0.660–0.853)	3/4–1	0.926	0.587	0.49	0.973	0.676
	T stage	0.726 (0.600–0.852)	T4b	0.519	0.933	0.737	0.843	0.824
	Location	0.696 (0.582–0.809)	Mid-thorax	0.778	0.613	0.885	0.42	0.657
	Deep ulcer	0.874 (0.782–0.966)	Yes	0.815	0.933	0.815	0.933	0.902
Validation cohort	Y1	0.841 (0.758–0.924)	2.1^a^	0.63	0.84	0.586	0.863	0.784
	Y2	0.917 (0.864–0.969)	2.58^a^	0.63	0.827	0.567	0.861	0.775
	Trachea invasion	0.524 (0.392–0.655)	Yes	0.154	0.893	0.333	0.753	0.696
	Tumor range	0.648 (0.531–0.745)	3/4–1	0.741	0.569	0.37	0.854	0.598
	T stage	0.562 (0.429–0.696)	T4b	0.778	0.893	0.429	0.761	0.716
	Location	0.584 (0.470–0.699)	Mid-thorax	0.63	0.587	0,354	0.815	0.598
	Deep ulcer	0.767 (0.649–0.885)	Yes	0.667	0.88	0.148	0.867	0.676

*Abbreviations*: *Y1* Joint predictive efficiency of quantitative CT analysis, *Y2* Joint predictive efficiency of quantitative CT analysis added with T stage, *AUC* Area under the curve, *SEN* Sensitivity, *SPE* Specificity, *PPV* Positive predict value, *NPV* Negative predict value, *ACU* Accuracy

^a^For quantitative parameters, the value >cutoff value indicated oesophageal fistula

### Performance of the combination of CT’s quantitative and qualitative analyses

Ulcer presence in tumor had the highest performance in predicting esophageal fistula with an AUC of 0.874 (95%CI 0.782–0.996), followed by tumor range with an AUC of 0.756 (95%CI: 0.660–0.853) and T stage with an AUC of 0.726 (95%CI: 0.600–0.852) (Table 3). However, only T stage was an independent factor (*p* = 0.024) in multivariate analysis. Therefore, T stage and quantitative analysis were combined to establish a model for predicting esophageal fistula in patients with esophageal cancer. The combination model (Y2 = 0.935*Y1 + 2.033* T-stage) for predicting esophageal fistula had AUCs of 0.953 and 0.917, and accuracies of 94.1% and 77.5% in primary and validation cohorts, respectively (Fig. 2, Table 3). Regarding to tumor stage, T1-4a was assigned as 0 and T4b was assigned as 1. However, there was no statistical significance between quantitative CT and T stage combination and quantitative CT analysis (*p* > 0.05).

### Clinical usefulness

**Fig. 4:**
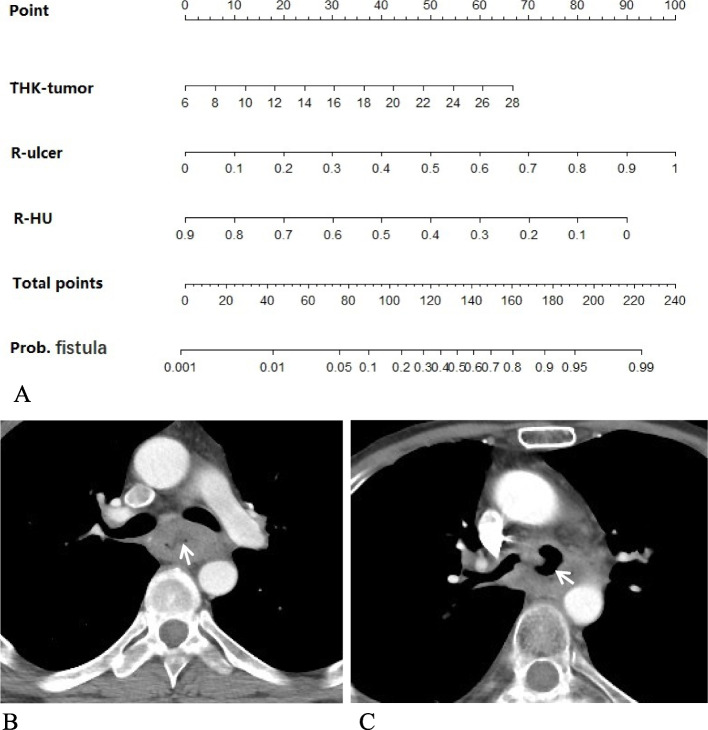
A nomogram established using quantitative CT parameters. **A** Nomogram presented a scoring model using three quantitative CT features, including tumor thickness (THK-tumor), the ulcer-to-tumor ratio (R-ulcer), and HU-min-to-HU-max ratio (R-HU). **B** Axial pre-therapy, contrast-enhanced MDCT showed an esophageal cancer with deep ulcer (arrow). The THK-tumor was 17 mm, corresponding to a point of 35 in nomogram. R-ulcer was 0.588(10 mm/17 mm), corresponding to a point of 60. R-HU was 0.35 (30HU/85HU), corresponding to a point of 55. The total points were 150, corresponding to the risk coefficient of 0.7, indicating the high risk of esophageal fistula. **C** Enhanced CT showed that the patient developed esophageal fistula after chemotherapy (arrow)

To facilitate clinical use, a nomogram based on quantitative CT parameters, including THK-tumor, R-ulcer and R-HU was developed (Fig. 4). The probability of fistula formation after treatment ranged from 0 to 1. A probability nearing 1 indicated high odds of esophageal fistula. Patients with esophageal cancer could benefit from this prediction model.

## Discussion

The diagnosis of esophageal fistula after treatment is challenging. Direct imaging methods, including esophagography, CT and esophagoscopy, could provide important clues for the diagnosis of esophageal fistula. Esophagography performed with water soluble agents could detect 75% of thoracic fistulas [21]. However, it may produce false-negative results in 10–38% of patients because of aspired hypertonic oral contrast solution, which may promote pulmonary edema [22]. Most surgeons are concerned about barium extravasation into the thorax [22, 23]. A recent study showed that oral meglumine diatrizoate esophagography using CT has a high sensitivity of 100% and a specificity of 98.9% in esophageal fistula screening [24]. However, in 20% of cases, the fistula could not be identified by preoperative CT. Indeed, water-soluble contrast agents are hyperosmolar and could draw fluid into the lungs, causing pulmonary edema in case of aspiration into the tracheobronchial tree [22]. Esophagoscopy both detects the fistula and helps determine the method of treatment [23]. On the other hand, small fistulas may even escape the sight of experienced endoscopists. In addition, endoscopy may make the fistula bigger and create more contaminations [21].

Therefore, predicting esophageal fistula occurrence and selecting high risk patients for fistula in esophageal SCC before treatment may change the treatment strategy and help prevent such a complication after treatment. Previous studies investigated clinical risk factors associated with esophageal fistula formation in esophageal SCC [20]. However, clinical risk factors predicting esophageal fistula showed moderate performance. In addition, no definitive factors in quantitative CT analysis have been identified in patients undergoing chemotherapy or CRT.

It was proved that T stage was a significant risk factor for fistula in accordance with previous results [25]. Another mentioned risk factor was deep ulcer presence and ulcer-to-tumor ratio in our study. Ulcerative lesions in esophageal carcinoma often destroy or penetrate the muscular layer, which may decrease the function of esophageal wall and increase the incidence rate of esophageal fistula in patients with increased pressure in the lumen because of swallowing or cough. Infection of ulcerative lesions also increases the risk of esophageal fistula. Sun et al. [26] found that 65% of patients (11/17) with deep ulcer in esophageal carcinoma developed esophageal fistula. Tsushima et al. [14] demonstrated that 89% of patients with esophageal fistula presented ulcerative esophageal carcinoma. Hu et al. [25] indicated that ulcerative esophageal cancer was associated with esophageal fistula. The advantage of this study was that quantitative CT analysis assessing deep ulcers was more accurate in predicting esophageal fistula.

This study showed that wall thickness in esophageal SCC was a significant risk factor for esophageal fistula formation. Wall thickness in esophageal SCC was associated with T stage. The current study also revealed that esophageal fistula was associated with deep ulcer presence and ulcer-to-tumor ratio. In the present study, there was a negative correlation between esophageal fistula and the ratio of minimum to maximum enhanced CT value. Tumor enhancement was positively correlated with density and micro-vessel structure in the tumor, which was the pathological basis of contrast-enhanced CT scanning [27]. Lower enhancement might reflect decreased intratumoral micro-vessel density, indicating less chance to absorb oxygen and nutrition as well as high odds of edema and necrosis. In the other study, there was a positive correlation between intratumoral micro-vessel count and desmoplasia [28]. Most esophageal carcinoma showed moderate enhancement, which was lower than that of the inflammatory or fibrotic component. Higher enhancement might reflect inflammatory or fibrotic changes or heterogeneity within the tumor. It was speculated that the tumor area with elevated enhancement might be the active area of fibrous tissue proliferation. We supposed that necrosis in the tumor showed lower enhancement with reduced CT value which might develop fistula easily, while elevated CT value with active desmoplasia might prevent fistula formation in the esophagus.

Consequently, we developed and validated a practical imaging scoring model using these quantitative CT parameters for predicting high risk of esophageal fistula before treatment. This imaging scoring model could provide an effective and handy tool for clinical strategy-making. In this study, the presence of deep ulcer was strongly correlated with R-ulcer with *Spearsman rho* of 0.984. Thus, when we explored for adding value of qualitative parameters to quantitative parameters, T-stage was selected for establishing Y2, while not the presence of deep ulcer. In the primary cohort, the combination of quantitative analysis and T stage only slightly improved the diagnostic performance of Y1 (AUC from 0.946 to 0.953). While in the validation cohort, we found that the combination model showed more stable diagnostic performance with AUC of 0.917. It suggested that the combination model may have a better prospect of clinical application. Surely it needs validation in large sample patients from other centers.

There were several limitations in this study. First, the sample size was small due to the low incidence of esophageal fistula. A much larger database from large multicenter trials might address this shortcoming and validate the reproducibility and generalization of this model. Secondly, this was a retrospective study, with the inherent selection bias. A well-designed prospective trial comparing esophageal fistula incidence between patients with esophageal SCC with or without risk factors before treatment is warranted. Thirdly, this prediction model did not completely mitigate the subjective evaluation of radiologists, and might be also affected by radiologist experience.

## Conclusion

We provide a handy and effective imaging model based on quantitative pretreatment CT parameters with excellent performance in predicting the risk of fistula formation in esophageal cancer patients treated with chemotherapy or CRT. This model offers an individualized assessment approach for esophageal SCC patients to guide clinical treatment for esophageal cancer with high risk of esophageal fistula.

## Supplementary Information

**Additional file 1: Supplementary Fig. 1.** Study flowchart. Abbreviations: SCC, esophageal squamous cell carcinoma; CRT, chemoradiotherapy.

**Additional file 2: Supplementary Table 1.** Characteristics of patients in the primary and validation cohorts. **Supplementary Table 2.** Intraclass correlation coefficients for radiological parameters.

## Data Availability

The data will not be shared because the ethics committee did not allow sharing of the data.

## Abbreviations

DEP-ulcer: Depth of deep ulcer
HU-max: Tumor maximum CT value
HU-min: Tumor minimum CT value
L-tumor: Tumor length
R-HU: HU-min-to-HU-max ratio
R-min: THK-min-to-THK-tumor ratio
R-residue: THK-residue-to-tumor ratio
R-ulcer: Ulcer-to-tumor ratio
THK-adjacency: Thickness of the lesion adjacent to the ulcer
THK-max: Thickness of the tumor on the maximum CT value layer
THK-min: Thickness of the tumor on the minimum CT value layer
THK-residue: Thickness of residual esophageal wall in the ulcer layer
THK-tumor: Tumor thickness
Y1: Joint predictive efficiency of quantitative CT analysis
Y2: Joint predictive efficiency of quantitative CT analysis combined with T stage
