# Supplementary figures and images for: Quantitative CT analysis to predict esophageal fistula in patients with advanced esophageal cancer treated by chemotherapy or chemoradiotherapy
Source: Cancer Imaging. 2022 Nov 4;22:62. doi: 10.1186/s40644-022-00490-2 (PMC9636691; doi:10.1186/s40644-022-00490-2)

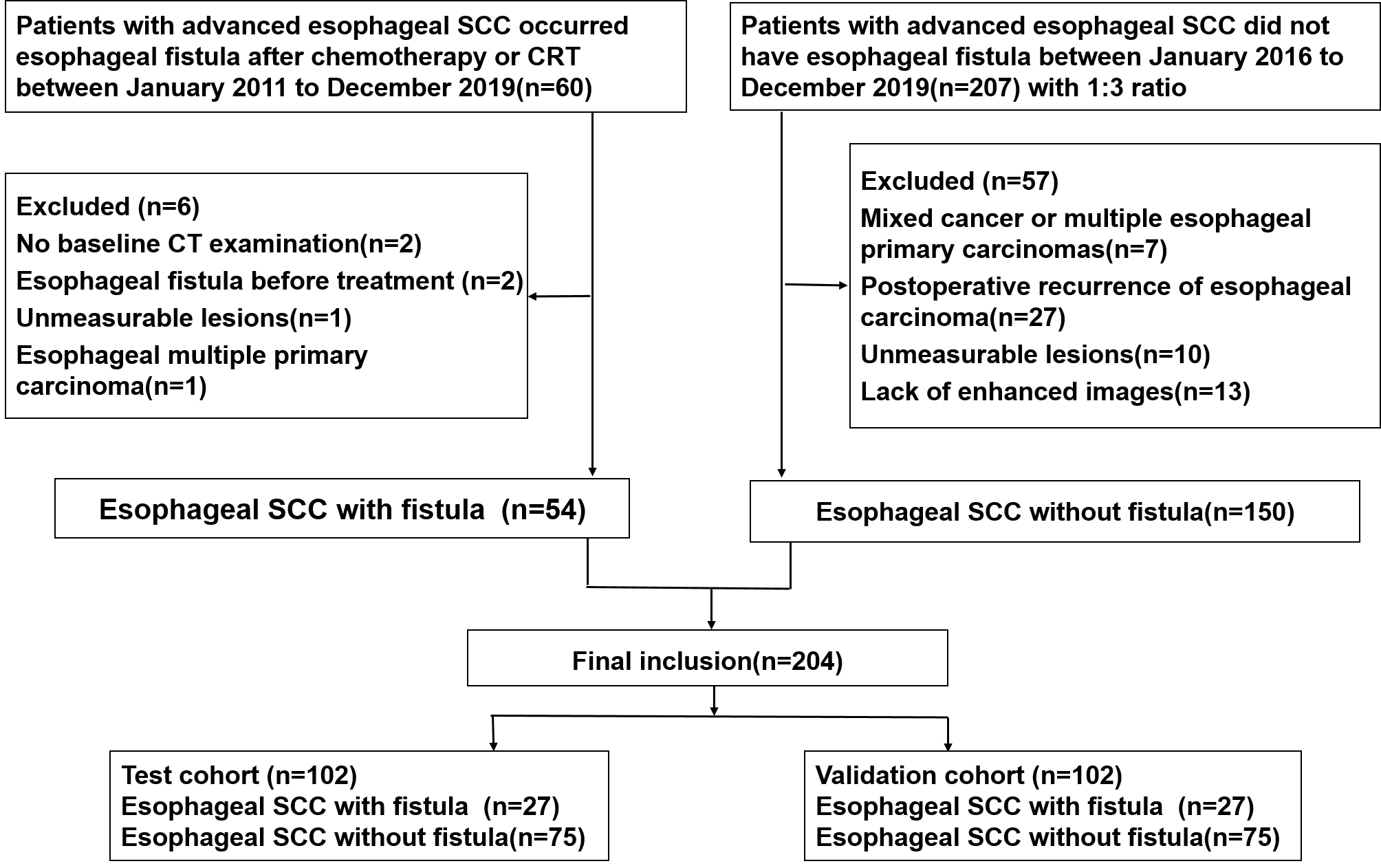

Supplement: Supplementary file 1 — Additional file 1: Supplementary Fig. 1. Study flowchart. Abbreviations: SCC, esophageal squamous cell carcinoma; CRT, chemoradiotherapy. [file 40644_2022_490_MOESM1_ESM.tif]
