# Supplementary material for: Quantitative CT analysis to predict esophageal fistula in patients with advanced esophageal cancer treated by chemotherapy or chemoradiotherapy
Source: Cancer Imaging. 2022 Nov 4;22:62. doi: 10.1186/s40644-022-00490-2 (PMC9636691; doi:10.1186/s40644-022-00490-2)
Supplement: Supplementary file 2 — Additional file 2: Supplementary Table 1. Characteristics of patients in the primary and validation cohorts. Supplementary Table 2. Intraclass correlation coefficients for radiological parameters. [file 40644_2022_490_MOESM2_ESM.docx]

**Supplementary Table 1. Characteristics of patients in the primary and validation cohorts**

| Characteristics |  | Primary cohort (n=102) | Validation cohort (n=102) | *P* |
| --- | --- | --- | --- | --- |
| Gender | Male | 95 | 92 | 0.447 |
|  | Female | 7 | 10 |  |
| Age, mean ± SD, years |  | 60.21±7.84 | 60.84±8.13 | 0.575 |
| Grade | Mid-high | 63 | 63 | / |
|  | Low | 39 | 39 |  |
| Location | Neck | 5 | 7 | 0.778 |
|  | Upper-thorax | 16 | 20 |  |
|  | Mid-thorax | 50 | 48 |  |
|  | Low-thorax | 31 | 27 |  |
| Treatment | Chemotherapy | 51 | 42 | 0.206 |
|  | Chemoradiotherapy | 51 | 60 |  |
| T stage | T1 | 0 | 0 | 0.46 |
|  | T2 | 9 | 8 |  |
|  | T3 | 73 | 66 |  |
|  | T4a | 1 | 4 |  |
|  | T4b | 19 | 14 |  |
| Trachea invasion | No | 88 | 89 | 0.836 |
|  | Yes | 14 | 12 |  |
| N stage | No | 5 | 8 | 0.39 |
|  | Yes | 97 | 94 |  |
| Metastasis | No | 84 | 86 | 0.707 |
|  | Yes | 18 | 16 |  |
| THK-tumor (mm) |  | 15.57±5.18 | 15.86±6.26 | 0.715 |
| L-tumor (mm) |  | 61.47±25.37 | 60.15±25.94 | 0.713 |
| Tumor range | 1-1/4 | 1 | 0 | 0.27 |
|  | 1/4-1/2 | 18 | 12 |  |
|  | 1/2-3/4 | 27 | 36 |  |
|  | 3/4-1 | 56 | 54 |  |
| Type | Focal | 20 | 26 | 0.315 |
|  | Diffuse | 82 | 76 |  |
| Luminal obliteration | No | 37 | 34 | 0.659 |
|  | Yes | 65 | 68 |  |
| Deep ulcer | No | 75 | 75 | / |
|  | Yes | 27 | 27 |  |
| DEP-ulcer (mm) | | 3.15±5.90 | 2.45±4.49 | 0.708 |
| THK-residue (mm) | | 12.38±6.18 | 12.79±7.27 | 0.948 |
| THK-adjacency (mm) | | 15.09±4.92 | 15.12±6.09 | 0.566 |
| R-ulcer(%) | | 21.52±42.05 | 16.93±30.23 | 0.741 |
| R-residue(%) | | 83.05±29.48 | 84.12±28.03 | 0.827 |
| HU-min (HU) | | 39.22±17.41 | 41.29±17.51 | 0.428 |
| THK-min (mm) | | 11.87±4.72 | 11.63±5.33 | 0.478 |
| R-min | | 0.77±0.18 | 0.75±0.19 | 0.333 |
| HU-max (HU) | | 92.90±13.91 | 91.99±15.24 | 0.477 |
| THK-max (mm) | | 11.87±4.28 | 11.18±3.56 | 0.442 |
| R-HU | | 0.43±0.18 | 0.45±0.17 | 0.318 |

**Abbreviations:** N stage, lymph node stage; THK-tumor, tumor thickness; L-tumor, tumor length; DEP-ulcer, depth of deep ulcer; THK-residue, thickness of residual esophageal wall in the ulcer layer; THK-adjacency, thickness of lesion adjacent to the ulcer; R-ulcer, ulcer-to-tumor ratio; R-residue, THK-residue-to-tumor ratio; HU-min, tumor minimum CT value; THK-min, thickness of the tumor on minimum CT value layer; R-min, THK-min-to-THK-tumor ratio; HU-max, tumor maximum CT value; THK-max, thickness of the tumor on maximum CT value layer; R-HU,HU-min-to-HU-max ratio

**Supplementary Table 2. Intraclass correlation coefficients for radiological parameters**

| Characteristics | ICC |
| --- | --- |
| T stage | 0.79 |
| Trachea invasion | 0.85 |
| N stage | 0.82 |
| Metastasis | 0.96 |
| THK-tumor (mm) | 0.96 |
| L-tumor (mm) | 0.94 |
| Tumor range | 0.83 |
| Type | 0.92 |
| Luminal obliteration | 0.87 |
| Deep ulcer | 0.85 |
| DEP-ulcer (mm) | 0.89 |
| THK-residue (mm) | 0.90 |
| THK-adjacency (mm) | 0.91 |
| HU-min (HU) | 0.88 |
| THK-min (mm) | 0.89 |
| HU-max (HU) | 0.92 |
| THK-max (mm) | 0.93 |

**Abbreviations:** N stage, lymph node stage; THK-tumor, tumor thickness; L-tumor, tumor length; DEP-ulcer, depth of deep ulcer; THK-residue, thickness of residual esophageal wall in the ulcer layer; THK-adjacency, thickness of lesion adjacent to the ulcer; HU-min, tumor minimum CT value; THK-min, thickness of the tumor on minimum CT value layer; HU-max, tumor maximum CT value; THK-max, thickness of the tumor on maximum CT value layer
